# Two Neonates with Congenital Hydrocolpos

**DOI:** 10.1155/2013/692504

**Published:** 2013-07-14

**Authors:** Vydehi Murthy, Jessica Costalez, Julie Weiner, Kristin Voos

**Affiliations:** Children's Mercy Hospital and Clinics, 2401 Gillham Road, Kansas City, MO 64108, USA

## Abstract

*Introduction*. Neonatal hydrocolpos is a rare condition. Hydrocolpos is cystic dilatation of the vagina with fluid accumulation due to a combination of stimulation of secretary glands of the reproductive tract and vaginal obstruction. The differential for a neonatal presentation of lower abdominal mass includes urogenital anomalies, Hirschsprung's, disease or sacrococcygeal teratoma. Prenatal diagnosis and early newborn imaging studies leads to early detection and treatment of these cases. *Case*. We report here two cases of neonatal hydrocolpos with prenatal diagnosis of lower abdominal mass. Postnatally, ultrasound, MRI imaging, and cystoscopy confirmed large cystic mass as hydrocolpos with distal vaginal obstruction. Both patients had enlarged renal system secondary to mass effect. *Conclusion*. High index of suspicion for hydrocolpos in a newborn presenting with fetal diagnosis of infraumbilical abdominal mass will facilitate timely intervention and prevention of complications.

## 1. Introduction

Congenital hydrocolpos is rare condition that can present as pelvic mass. The purpose of this report is to increase the index of suspicion for hydrocolpos in patients with prenatal ultrasound (US) diagnosis of pelvic mass and to describe how early diagnosis and management in neonatal period can improve outcome. 

Hydrocolpos is vaginal distension with fluid accumulation due to a combination of stimulated secretory glands and vaginal obstruction [1]. The fluid accumulation can also be due to the presence of urogenital sinus with collection of urine. Complications like hydronephrosis and gastrointestinal obstruction secondary to mass effect make it imperative that these infants be evaluated and treated soon after birth.

## 2. Case Report 

*Case  1*. Neonate A is a female infant who had a fetal US at 30 weeks of gestation significant for an abdominal mass. She was delivered at 37 weeks via c-section due to macrosomia. Genital examination revealed a morphologically normal female with no dilation of the hymenal rim. A 5-French feeding tube was passed into the vagina approximately 2 cm and no further. Plain abdominal radiograph showed an opaque mass in the mid lower abdomen with a mass effect displacing the bowels. Pelvic US showed a cystic mass in the lower pelvis with a fluid-debris level. The large mass displaced the urinary bladder anteriorly and did not involve the uterus. She also had left sided hydronephrosis secondary to compression of the proximal ureter by the mass. MRI of pelvis confirmed hydrocolpos with narrowing of the distal vagina down to the perineum. Interventional radiologist drained the cystic mass transabdominally and aspirated milky fluid. The laboratory results showed that aspirated fluid was not consistent with urine. She had resolution of the left sided hydronephrosis with minimal fluid in vaginal vault prior to discharge home at 4 weeks of age. The infant was followed by gynecologist and had serial US which showed gradual accumulation of the fluid in the vaginal vault. 

**Figure 1:**
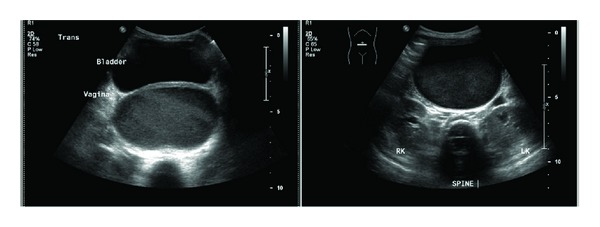
Pelvic ultrasound of Neonate A showing fluid collection in both bladder and vagina.

She was admitted at 10 months of age for fever, distended abdomen, and urinary retention (Figure 1). Interventional radiologist aspirated pus transabdominally and placed a vaginostomy tube that was left in place to continue to drain the vaginal vault until surgery. The fluid from the pyocolpos grew *E. coli* and the infant was treated with antibiotics. After completing antibiotic course, she had definitive reconstructive therapy with pull though vaginoplasty. Cystovaginoscopy showed that the vaginoscopy tube that was placed to drain had traversed the bladder essentially creating a vesicovaginal fistula. Both the vagina and bladder were repaired with widely patent perineal vagina and recovered well.

**Figure 2:**
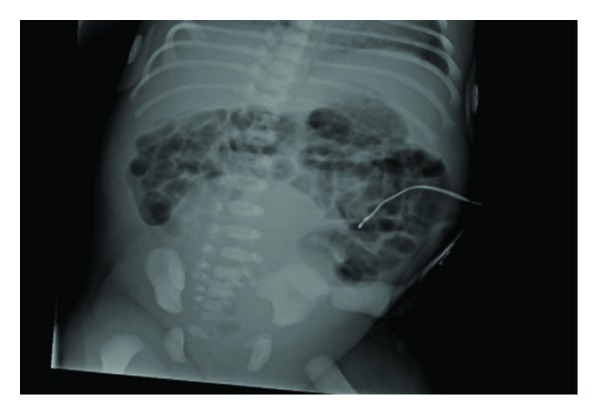
Plain abdominal radiograph of Neonate B showing infraumbilical midline abdominal opaque mass.

**Figure 3:**
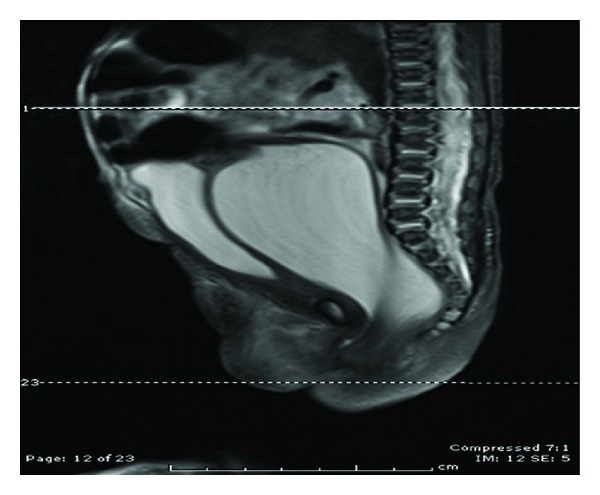
Neonate B: Sagittal T2W image through the abdomen and pelvis demonstrates a fluid-distended vagina between urinary bladder and compressed rectum. The distal vaginal canal is abnormally narrow with only a subtle channel identified. Suspicious for vaginal stenosis versus transverse muscular septum given the soft tissue signal which is isointense to muscle.

*Case  2*. Neonate B is a 34-weeks premature female infant who had a fetal US diagnosis of abdominal mass at 31 weeks of gestation. The infant was delivered by c-section for concern of increasing mass size and the pelvic dilation of both kidneys. External genitalia appeared normal with no labial enlargement. There was mild bulging of the vaginal introitus. An attempt to penetrate the hymenal membrane with 5-French catheter was unsuccessful. Plain abdominal radiograph showed an infraumbilical midline opaque mass (Figure 2). An abdominal US reported a cystic mass with bilateral pelvic dilation consistent with bladder outlet obstruction and ascites. MRI findings suggested lower vaginal atresia with accumulation of fluid, normal appearing uterus, and cervix with atretic vaginal canal (Figure 3). Interventional radiologist performed US-guided drainage of the vagina. The fluid showed elevated creatinine level suggesting that it was urine. Cystovaginoscopy further clarified her anatomy of distal vaginal atresia and a confluence between the vagina and urinary tract distal to the urinary sphincter. The final diagnosis was hydrocolpos with urogenital sinus anomaly. A transabdominal vaginostomy tube was placed to continually drain urine that accumulated in the vagina and discharge home after educating the mother on vaginostomy tube care. Renal US showed that bilateral hydronephrosis had completely resolved at time of discharge home. At 8 months of age, she underwent a repair using a partial urogenital sinus mobilization technique. The area of the fistula was identified and transected and the urethra vagina both repaired in offset fashion. She underwent a vagina pull down to the perineal skin and recovered well.

## 3. Discussion

We report here 2 cases presented with prenatal ultrasound diagnosis of lower abdominal mass. The final diagnosis was hydrocolpos secondary to distal vaginal atresia. The infant in the second case also had congenital urovaginal sinus.

Congenital hydrocolpos is an uncommon disorder characterized by vaginal distension with fluid accumulation. It is believed to be due to increased secretion by cervical mucous glands secondary to maternal hormone stimulation which gradually accumulates, expands and builds up into a pelvic mass due to vaginal outlet obstruction [2, 3].

Hydrocolpos can be associated with genitourinary anomalies from persistent urogenital sinus to cloacal dysgenesis. A detailed prenatal US focusing on the fetal pelvic anatomy will provide anatomic details and facilitate appropriate prenatal counseling to parents [4]. Early US diagnosis improves the prognosis especially in children with associated complication of obstructive uropathy [5]. The initial diagnosis of pelvis mass in both of our patients was done by prenatal US. Both parents were offered counseling by a perinatlogist and neonatologist. Our patients did not have fetal MRI but it can be useful in further differentiating pelvic mass [6]. MRI imaging provides additional anatomic details with excellent soft tissue contrast to determine the thickness of the transverse septum, length of the atresia, and the presence or absence of a cervix [7, 8].

Vaginal atresia can be associated with several syndromes like Mckusick-Kaufman syndrome and Bardet-Biedl syndrome. Vaginal septum can also be associated with syndromes associated with mullerian aplasia. Both of our patients were eumorphic with no associated congenital anomalies. They both had normal uterus and fallopian tubes but ovaries were not visualized on imaging studies. There were no family history of urogenital disorders and no genetic testing was performed. 

The most common complication of hydrocolpos is compression of the bladder, leading to hydronephrosis, which can ultimately cause kidney damage [9]. Hammad and Upadhyay reported that hydrometrocolpos was the cause in 23% of patients with infravesical obstruction and 39% of those cases presented at birth as a result of prenatal ultrasound diagnosis of pelvic pathology [10]. Bischoff et al. reviewed retrospectively 411 medical records of patients diagnosed with cloacal anomalies of which about 28% had an associated hydrocolpos [9]. Other complications including sepsis and pyocolpos have been reported in the literature [9, 11, 12]. Infants with prenatal diagnosis of pelvic mass should be categorized as high risk deliveries and should be promptly followed by multidisciplinary team soon after birth.

Complications of hydrocolpos can be decreased by timely intervention and drainage of accumulated fluid. The drainage of hydrocolpos dramatically improved the hydronephrosis in both of our patients; both of our patients also had reaccumulation of fluid in vaginal vault. Such patients benefit from draining the hydrocolpos with an indwelling transabdominal vaginostomy tube for continuous drainage until the definitive repair. The drainage is performed by interventional radiologist transabdominally under US which enables real-time evaluation without radiation exposure [13]. The transabdominal drainage of hydrocolpos with indwelling tube is more preferred than transvaginal drainage to prevent reaccumulation [9]. In general, infants with hydrocolpos and urogenital sinus have increased risk of sepsis due to collection of urine in vaginal vault. There have been reported deaths due to sepsis associated with hydrocolpos [11, 12].

## 4. Conclusion

Hydrocolpos is a rare condition in the neonate and should be suspected when a prenatal US identifies a midline abdominopelvic mass. Prenatal diagnosis and early newborn imaging lead to early detection and treatment of these cases. This can prevent complications secondary to compression and obstruction of surrounding structures.
